# Effect of Hypoglycemia on Inflammatory Responses and the Response to Low-Dose Endotoxemia in Humans

**DOI:** 10.1210/jc.2018-01168

**Published:** 2018-09-24

**Authors:** Ahmed Iqbal, Lynne R Prince, Peter Novodvorsky, Alan Bernjak, Mark R Thomas, Lewis Birch, Danielle Lambert, Linda J Kay, Fiona J Wright, Ian A Macdonald, Richard M Jacques, Robert F Storey, Rory J McCrimmon, Sheila Francis, Simon R Heller, Ian Sabroe

**Affiliations:** 1 Department of Infection, Immunity and Cardiovascular Disease, University of Sheffield, Sheffield, United Kingdom; 2 Sheffield Teaching Hospitals NHS Foundation Trust, Sheffield, United Kingdom; 3 Department of Oncology and Metabolism, University of Sheffield, Sheffield, United Kingdom; 4 INSIGNEO Institute for In Silico Medicine, University of Sheffield, Sheffield, United Kingdom; 5 Institute of Cardiovascular Sciences, University of Birmingham, Birmingham, United Kingdom; 6 MRC/ARUK Centre for Musculoskeletal Ageing Research, National Institute for Health Research Nottingham Biomedical Research Centre, Nottingham, United Kingdom; 7 Division of Physiology, Pharmacology and Neuroscience, School of Life Sciences, University Nottingham, Nottingham, United Kingdom; 8 School of Health and Related Research, University of Sheffield, Sheffield, United Kingdom; 9 Division of Molecular and Clinical Medicine, University of Dundee, Dundee, United Kingdom

## Abstract

**Context:**

Hypoglycemia is emerging as a risk for cardiovascular events in diabetes. We hypothesized that hypoglycemia activates the innate immune system, which is known to increase cardiovascular risk.

**Objective:**

To determine whether hypoglycemia modifies subsequent innate immune system responses.

**Design and Setting:**

Single-blinded, prospective study of three independent parallel groups.

**Participants and Interventions:**

Twenty-four healthy participants underwent either a hyperinsulinemic-hypoglycemic (2.5 mmol/L), euglycemic (6.0 mmol/L), or sham-saline clamp (n = 8 for each group). After 48 hours, all participants received low-dose (0.3 ng/kg) intravenous endotoxin.

**Main Outcome Measures:**

We studied *in-vivo* monocyte mobilization and monocyte-platelet interactions.

**Results:**

Hypoglycemia increased total leukocytes (9.98 ± 1.14 × 10^9^/L vs euglycemia 4.38 ± 0.53 × 10^9^/L, *P* < 0.001; vs sham-saline 4.76 ± 0.36 × 10^9^/L, *P* < 0.001) (mean ± SEM), mobilized proinflammatory intermediate monocytes (42.20 ± 7.52/μL vs euglycemia 20.66 ± 3.43/μL, *P* < 0.01; vs sham-saline 26.20 ± 3.86/μL, *P* < 0.05), and nonclassic monocytes (36.16 ± 4.66/μL vs euglycemia 12.72 ± 2.42/μL, *P* < 0.001; vs sham-saline 19.05 ± 3.81/μL, *P* < 0.001). Following hypoglycemia vs euglycemia, platelet aggregation to agonist (area under the curve) increased (73.87 ± 7.30 vs 52.50 ± 4.04, *P* < 0.05) and formation of monocyte-platelet aggregates increased (96.05 ± 14.51/μL vs 49.32 ± 6.41/μL, *P* < 0.05). Within monocyte subsets, hypoglycemia increased aggregation of intermediate monocytes (10.51 ± 1.42/μL vs euglycemia 4.19 ± 1.08/μL, *P* < 0.05; vs sham-saline 3.81± 1.42/μL, *P* < 0.05) and nonclassic monocytes (9.53 ± 1.08/μL vs euglycemia 2.86 ± 0.72/μL, *P* < 0.01; vs sham-saline 3.08 ± 1.01/μL, *P* < 0.05), with platelets compared with controls. Hypoglycemia led to greater leukocyte mobilization in response to subsequent low-dose endotoxin challenge (10.96 ± 0.97 vs euglycemia 8.21 ± 0.85 × 10^9^/L, *P* < 0.05).

**Conclusions:**

Hypoglycemia mobilizes monocytes, increases platelet reactivity, promotes interaction between platelets and proinflammatory monocytes, and potentiates the subsequent immune response to endotoxin. These changes may contribute to increased cardiovascular risk observed in people with diabetes.

## Teaser

Using an in vivo human experimental model, we show that hypoglycemia primes the innate immune system, leading to a more profound inflammatory response to a subsequent inflammatory stimulus.

Hypoglycemia is associated with a greater propensity to adverse cardiovascular (CV) outcomes in diabetes (1–3). To determine whether such outcomes were dependent on changes in innate immune responses, we devised a model whereby subjects were challenged with a hypoglycemic clamp, and then the durable effects on the innate immune system were probed by an *in vivo* endotoxin challenge 48 hours later.

Iatrogenic hypoglycemia remains a major barrier to effective treatment of insulin-treated diabetes (4). The Action to Control Cardiovascular Risk in Diabetes trial showed that intensive glucose control, during which patients were exposed to significantly more hypoglycemia (5), was associated with excess CV mortality. Despite the evidence confirming an association between hypoglycemia and mortality, cause and effect has not been established. Trial evidence suggests that the relationship is, at least in part, explained by confounding, that is, that hypoglycemia identifies patients with comorbidities who are both vulnerable to hypoglycemia and more likely to die for other reasons (6). Nevertheless, a recent large meta-analysis (7) suggested that comorbidities alone are unlikely to explain this relationship. Furthermore, there is a growing body of evidence highlighting a number of mechanisms whereby hypoglycemia may lead to CV events (8, 9).

Hypoglycemia has proinflammatory consequences, including increases in levels of factor VIII and von Willebrand factor and impaired fibrinolysis (10–12). In addition, hypoglycemia has been shown to increase proinflammatory cytokines (12–14) and promote rises in the levels of proatherogenic cell adhesion molecules (12). Repeated episodes of hypoglycemia have also been reported to impair nitric oxide–mediated vasodilation (15).

Monocytes are phagocytes that are central to the etiology of atherosclerosis (16) and play a role in precipitating acute CV events by promoting plaque destabilization and rupture (17). The extent to which monocytosis and monocyte activation are modified by hypoglycemia remains uncertain. Recent studies have also determined that monocytes can be classified into three distinct subsets, called classic monocytes (CMs: CD14^++^ CD16^−^, and Mon1), intermediate monocytes (IMs: CD14^++^ CD16^+^, Mon2), and nonclassic monocytes (NCMs: CD14^+^ CD16^++^, Mon3) (18, 19). A number of observational studies indicate that IMs may be particularly proatherogenic. Elevated levels of IMs are associated with adverse CV outcomes (20–23), independently predict future CV events (22), and have been associated with coronary plaque vulnerability in patients with angina (24). Elevated levels of CMs may also independently predict CV events (25).

Acute myocardial infarction results in monocytosis, mediated by sympathetic nervous system activation (26). In humans, CD16^+^ monocytes selectively mobilize, in a catecholamine-dependent fashion, after exercise (27). Because epinephrine is the key counterregulatory hormone produced in response to hypoglycemia, we hypothesized that hypoglycemia would also exert significant effects on monocytes. We further hypothesized that we would see additional synergistic changes in monocyte and platelet activation, as revealed by formation of monocyte-platelet aggregates (MPAs), which are increased after acute myocardial infarction (20, 28). In large prospective studies, CV events did not appear to occur during the hypoglycemic episode *per se,* but there was an increased risk of events in the weeks and months after the episode (29–31). Therefore, we hypothesized that acute hypoglycemia may prime the innate immune system, leading to a more pronounced inflammatory response to a subsequent inflammatory stimulus downstream from the initial episode of hypoglycemia. It is also relevant to note that people with diabetes experience increased incidences of acute and chronic infections that will further activate innate immunity. To reveal whether hypoglycemia modulated monocyte function in the human *in vivo,* we chose to combine a classic hypoglycemic stimulus with a subsequent *in vivo* systemic stimulus of the innate immune system. To do so, we combined hyperinsulinemic-hypoglycemic, euglycemic, and sham-saline clamps with low-dose intravenous endotoxin challenge 48 hours later in healthy participants. Endotoxin, otherwise known as gram-negative bacterial lipopolysaccharide, was used because it induces a short-lived, sterile inflammation that is both safe and reproducible (32).

## Materials and Methods

### Study design and participants

This was a single-blinded, prospective study of three independent parallel groups (hyperinsulinemic-hypoglycemia, euglycemia, and sham-saline controls) conducted in a random group order at the Clinical Research Facility, Northern General Hospital, Sheffield, United Kingdom between January 2015 and April 2016. We therefore had three groups that had euglycemia with insulin, hypoglycemia with insulin, or saline. Each then received endotoxin. Baseline values at the start of endotoxin administration were studied in all groups, providing a set of data obtained before endotoxin. A total of 24 healthy participants without diabetes were recruited from the University of Sheffield and Sheffield Teaching Hospitals, with written informed consent in accordance with a protocol approved by Yorkshire and the Humber-Sheffield Research Ethics Committee (REC 14/YH/1264). All participants had a HbA1c <6.5% (<48 mmol/mol), measured with ion exchange high-performance liquid chromatography, and none had impaired glucose tolerance based on HbA1c as judged by the American Diabetes Association criteria (33, 34). Participants were in good health, as determined by a medical history, physical examination, vital signs, and clinical laboratory test results including full blood count and renal and liver function. Those with an intercurrent illness in the previous 4 weeks were excluded. Participants taking beta-blockers, QT interval–prolonging agents, and anticoagulant, antiplatelet, or anti-inflammatory medications were also excluded. Female participants were on secure contraception and also had negative urinary pregnancy tests on the morning of the clamp and endotoxin studies.

### Clamp studies

All participants attended at 0800 hours after an overnight fast and were blinded to their group allocation. Participants were instructed to avoid caffeine, alcohol, and vigorous exercise 24 hours before the study visit. An intravenous cannula was inserted into the antecubital fossa of the nondominant arm for insulin and dextrose infusion. A second intravenous cannula was inserted into the antecubital fossa of the dominant arm for all blood measurements except glucose. After application of a local anesthetic cream (lidocaine/prilocaine; Astra-Zeneca, Macclesfield, UK) to the dorsal hand or wrist of the nondominant arm, a retrograde cannula was inserted and the hand placed in a warming chamber (The Sheffield Hand Warmer, Sheffield, UK) at 55°C to allow arterialization of venous blood for glucose measurement. In the hypoglycemia and euglycemia study groups, a primed continuous insulin (Human Actrapid; Novo Nordisk Pharmaceuticals LT, Crawley, UK) infusion was administered at a rate of 90 mU/m^2^/min, with total insulin exposure matched between groups. A 20% dextrose (Baxter Healthcare Ltd., Thetford, UK) variable-rate infusion was administered simultaneously and the rate adjusted according to arterialized whole blood glucose concentrations measured every 5 minutes with a glucose oxidase method (Yellow Springs Instrument 2300 STAT, Yellow Springs, Ohio). After a brief (30-minute) euglycemic phase in both groups, blood glucose was lowered to 2.5 mmol/L in the hypoglycemia group and maintained for 60 minutes at this level. In the euglycemia group, blood glucose was maintained at 6 mmol/L for 60 minutes. Participants in the sham-saline group were investigated identically but did not receive insulin/dextrose infusions and instead received a slow intravenous infusion of 0.9% NaCl (Baxter, Baxter Healthcare Ltd.) at a predetermined fixed rate. Thus, participants in the sham-saline group were under normoglycemic conditions, allowing us to control for the effects of insulin and dextrose. Blood was sampled at baseline and at 60 minutes. Members of staff processing assays were blinded to glucose group allocation.

### Endotoxin challenge

Endotoxin challenge is a safe and well-studied model of innate immune activation *in vivo* (35). Forty-eight hours after the clamp, participants reattended at 0800 hours, having fasted overnight and refrained from caffeine, alcohol, and vigorous exercise since the clamp visit. An intravenous cannula was inserted into the antecubital fossa of the nondominant arm for administration of endotoxin and a second cannula inserted into the contralateral antecubital fossa for blood sampling. All participants received 0.3 ng/kg *Escherichia coli* O:113 lipopolysaccharide (Clinical Centre Reference Endotoxin; National Institutes of Health, Bethesda, MD). Endotoxin powder was reconstituted in 1 mL sterile 0.9% NaCl to form a solution at a concentration of 1000 ng/mL, which was vortexed for 60 minutes. The weight-adjusted dosage of endotoxin was obtained from this solution, added to 5 mL of 0.9% NaCl, and administered as a slow bolus injection over 1 minute. An intravenous infusion of 500 mL of 0.9% NaCl (Baxter Healthcare Ltd.) then continued for 4 hours after endotoxin to avoid hypotension. Venous blood was sampled at baseline and 2, 4, and 6 hours after endotoxin. All laboratory measurements were performed by staff blinded to glucose group allocation.

### Biochemical analysis

To measure epinephrine, venous forearm blood was collected into chilled lithium heparin tubes and centrifuged at 4°C, 1000*g* for 10 minutes. The resulting supernatant was stored at −80°C until assayed by high-performance liquid chromatography. To determine insulin levels, EDTA-anticoagulated blood was centrifuged at 3000*g* for 10 minutes, and free insulin levels were measured in the resulting plasma with an immunoassay (Roche Cobas; Roche Diagnostics, Burgess Hill, West Sussex, UK). Venous blood was centrifuged at 3000*g* for 10 minutes, and the resulting serum was used to measure cortisol and GH with an immunoradiometric assay (Roche Cobas). Sample collection for cortisol and GH was controlled for time of day across the three study groups.

### Cell counts and flow cytometry

Total and differential white blood cell (WBC) and platelet counts in EDTA-anticoagulated blood were determined with an automated clinical grade Sysmex cell counter (XN-9000; Sysmex, Milton Keynes, UK). For the clamp visit, alternative WBC counting methods were piloted for the first two subjects in each group, but these were later deemed less accurate than the Sysmex cell counter. Data shown in Figs. 2, 3, and 4a, 4c, 4d, 4e, and 4f are Sysmex data from n = 6 in each study group. Flow cytometry was used to determine MPAs: blood was collected into tubes containing trisodium citrate dihydrate (3.13% w/v) and incubated in a heat block at 37°C for 10 minutes; erythrocytes were lysed with fluorescence-activated cell sorting (FACS) lyse solution (BD, Oxford, UK) and stained with fluorescein isothiocyanate–conjugated CD16 (BioLegend, London, UK), allophycocyanin-conjugated CD14 (BioLegend), and phycoerythrin (PE)-conjugated CD42a (BD) in addition to matched isotype controls. Cells were fixed with FACS Fix (BD) and analyzed with flow cytometry (Accuri C6 multicolor flow cytometer; BD) within a consistent time frame for all subjects. Monocytes were gated based on morphology and CD14 expression. Neutrophils were gated on morphology and through exclusion of monocytes. Monocyte-platelet aggregation was determined by measuring monocyte mean fluorescence of the platelet-specific marker CD42a. To phenotype and enumerate monocyte subsets, anticoagulated blood was stained with fluorescein isothiocyanate–conjugated CD16 (BioLegend), allophycocyanin-conjugated CD14 (BioLegend), PE-conjugated CD66c (BD), PE-Cy7-conjugated CD11b (BioLegend), and (PerCP)-eFluor® 710-conjugated CX_3_C chemokine receptor 1 (CX3CR1; eBioscience, Altrincham, UK). Matched isotype controls and a “fluorescence minus one” strategy optimized compensation. Stained whole blood was lysed with FACS lyse as above and the pellet resuspended in PBS before fixation with 1% w/v formaldehyde. Samples were immediately processed for analysis with flow cytometry (LSRII; BD). Monocytes were gated based on morphological characteristics and through the exclusion of neutrophils with CD66c. Monocyte subsets were identified based on relative expression of CD14 and CD16. Flow cytometry data were analyzed with FlowJo version 10 (FlowJo LLC, Ashland, OR).

### Platelet aggregation

Platelet aggregation was measured with impedance aggregometry (Multiplate®; Verum Diagnostica GmBH, Munich, Germany). Aliquots of 300 μL saline and 300 μL hirudin-anticoagulated blood were added to the cuvette and incubated at 37°C for 3 minutes. Then 20 μL ADP (at a final concentration of 6.45 μM) was added as agonist, and the assay commenced. The area under the curve was measured, which represents the level of platelet aggregation.

### Statistical analysis

Our pilot data indicated that a sample size of seven participants per group would have 90% power to detect a 50% relative difference in mobilization of monocytes between hypoglycemia and controls. Eight subjects were recruited per group to allow for a 13% dropout rate. Mean baseline measurements of glucose were compared between groups with ANOVA. Mean measurements of glucose, insulin, and epinephrine at 60 minutes after clamp were compared, adjusting for clamp baseline measurement, with analysis of covariance. In the event of unequal variance between groups, a log transform was applied and the analysis repeated. Longitudinal and between-group comparisons were made for postendotoxin measurements with mixed effects linear models. For models examining between-group differences, the baseline endotoxin measurement was included as a covariate. For all mixed-effects linear models, an autoregressive correlation structure was used to allow for the correlation between multiple measurements on the same person. Planned contrasts were made with baseline and between groups at equivalent time points with Sidak correction for multiple comparisons. All data are expressed as mean ± SEM, unless otherwise specified, and a *P* < 0.05 was deemed statistically significant. Analysis was performed in SPSS version 22.0 (IBM, Chicago, IL).

## Results

### Participants

Study participants across the three groups were well matched for age, sex, body mass index, HbA1c, and total WBC count, with no significant differences at screening (Table 1). Participant numbers at each stage of study are illustrated in a flow diagram (Supplemental Flow Diagram).

**Table 1.** Comparison of Participant Characteristics at Baseline

Parameter	Hypoglycemia	Euglycemia	Sham-Saline	*P*
Total (n = 24)	8	8	8	N/A
Sex, M/F	4/4	4/4	4/4	N/A
Age, y	21 (19–22)	21 (20–23)	21.5 (21–26)	0.299
Body mass index, kg/m^2^	24 ± 2	23 ± 2	24 ± 4	0.638
HbA1c				
%	5.2 ± 0.31	5.2 ± 0.26	5.1 ± 0.14	0.792
mmol/mol	34 ± 3.6	33.5 ± 2.8	32.6 ± 1.4	0.616
Total WBCs, ×10^9^/L	6.26 ± 1.42	4.83 ± 0.91	4.50 ± 1.69	0.102

Data are mean ± SD or median (interquartile range). *P* values indicate comparisons between study groups via parametric or nonparametric testing.

### Clamp studies

#### Glucose, insulin, and counterregulatory hormones

**Figure 1.**
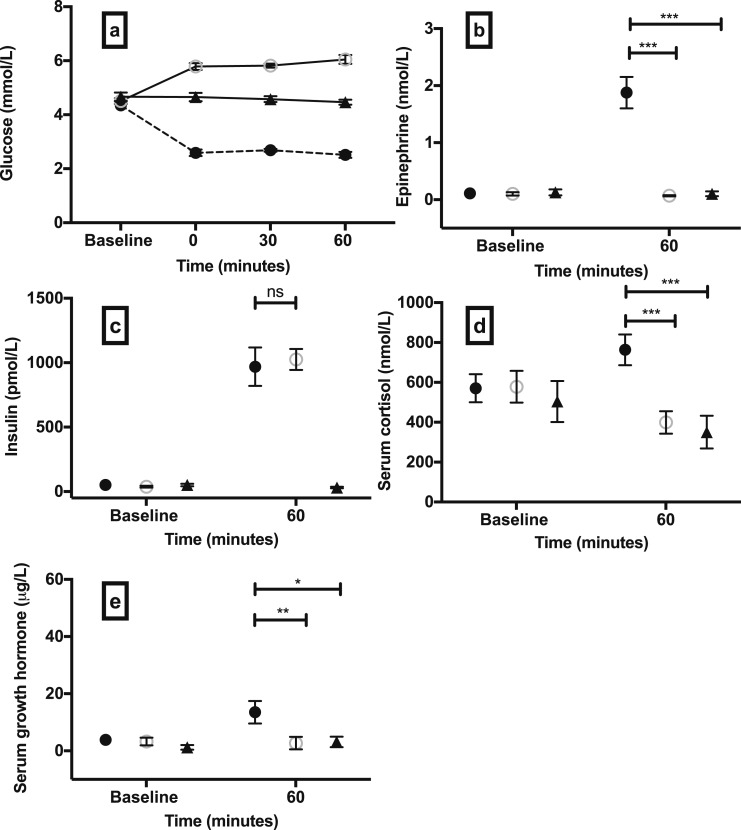
Glucose, insulin, and counterregulatory hormones in clamp studies. (a) Arterialized whole blood glucose values during hyperinsulinemic hypoglycemic, euglycemic, and sham-saline clamps, (b) epinephrine, (c) free insulin, (d) cortisol, and (e) GH values after 60 min of hypoglycemia, euglycemia, or sham-saline injection. Data are mean (SEM). *P < 0.05; **P < 0.01; ***P < 0.001; ns-nonsignificant; *P* values are provided for comparison between study groups. Black circles (and dashed line 1a), hypoglycemia group; open circles, euglycemia group; black triangles (and solid line 1a), sham-saline group.

Arterialized blood glucose values are shown in Fig. 1a. The glucose values were 2.51 ± 0.11 mmol/L and 6.04 ± 0.16 mmol/L at the end of the hypoglycemia and euglycemia clamps, respectively. Glucose values at the end of the sham-saline clamp were 4.64 ± 0.09 mmol/L. A counterregulatory response to hypoglycemia was evident, with epinephrine levels during hypoglycemia (1.87 ± 0.25 nmol/L) being significantly higher (*P* < 0.001) than those during euglycemia (0.07 ± 0.01 nmol/L) and sham-saline (0.10 ± 0.04 nmol/L) (Fig. 1b). Free insulin levels at the end of clamp were similar between the hypoglycemia (968.5 ± 149.1 pmol/L) and euglycemia groups (1025.4 ± 81.4 pmol/L, *P* = 0.996) but significantly higher (*P* < 0.001) than those in the sham-saline (31.3 ± 6.3 pmol/L) group (Fig. 1c). Serum cortisol and GH were significantly higher in the hypoglycemia group than in the euglycemia and sham-saline groups (Fig. 1d and 1e).

#### Total and differential leukocyte count

**Figure 2.**
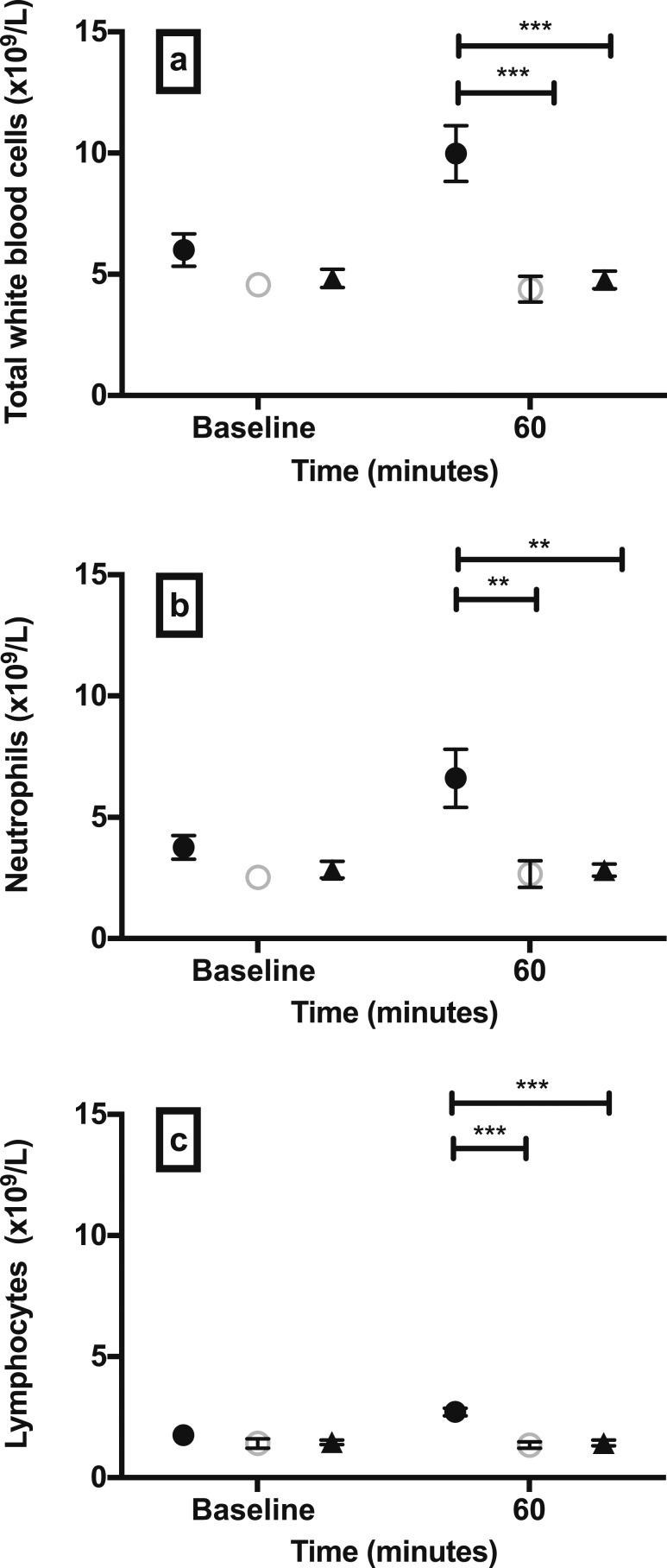
Peripheral total WBC, neutrophil, and lymphocyte kinetics in experimental hypoglycemia and controls. Number of circulating (a) total WBCs, (b) neutrophils, and (c) lymphocytes after 60 min of hypoglycemia, euglycemia, or sham-saline injection. Data are mean (SEM). **P < 0.01; ***P < 0.001; *P* values are provided for comparison between study groups. Black circles, hypoglycemia group; open circles, euglycemia group; black triangles, sham-saline group.

**Figure 3.**
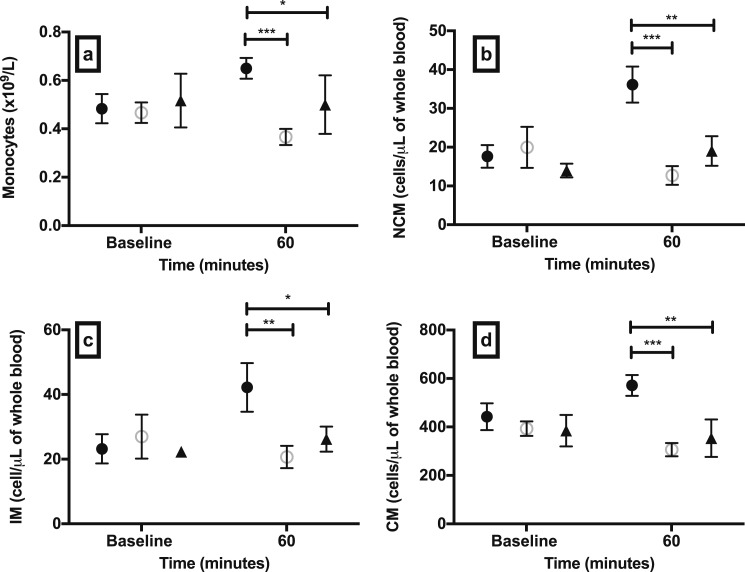
Total monocyte count and monocyte subset kinetics in experimental hypoglycemia groups and controls. Absolute circulating numbers of (a) total monocytes and monocyte subsets (b) NCM, (c) IM, and (d) CM after 60 min of hypoglycemia, euglycemia, or sham-saline injection. Data are mean (SEM). *P < 0.05; **P < 0.01; ***P < 0.001; *P* values are provided for comparison between study groups. Black circles, hypoglycemia group; open circles, euglycemia group; black triangles, sham-saline group.

We determined whether hypoglycemia results in changes in circulating leukocytes. Hypoglycemia significantly increased the total number of WBCs compared with controls (Fig. 2a). There was an increase across all classes of leukocytes studied, including neutrophils (Fig. 2b), lymphocytes (Fig. 2c), and total monocytes (Fig. 3a).

#### Monocyte subsets

We sought to determine whether hypoglycemia exerted specific effects on monocyte subsets associated with cardiac pathology. Hypoglycemia increased the absolute number of all three circulating monocyte subsets compared with euglycemia and sham-saline (Fig. 3b–3d). The number of circulating NCMs after 60 minutes of hypoglycemia compared with baseline (17.6 ± 2.9 cells/μL) increased twofold. IM numbers after 60 minutes of hypoglycemia compared with baseline (23.2 ± 4.5 cells/μL) increased by a factor of 1.81, and CMs after 60 minutes of hypoglycemia compared with baseline (442.4 ± 55.3 cells/μL) increased by a factor of 1.29. There were no significant differences in the baseline values of all three monocyte subsets between the study groups.

#### Platelet count, aggregation, and MPAs

**Figure 4.**
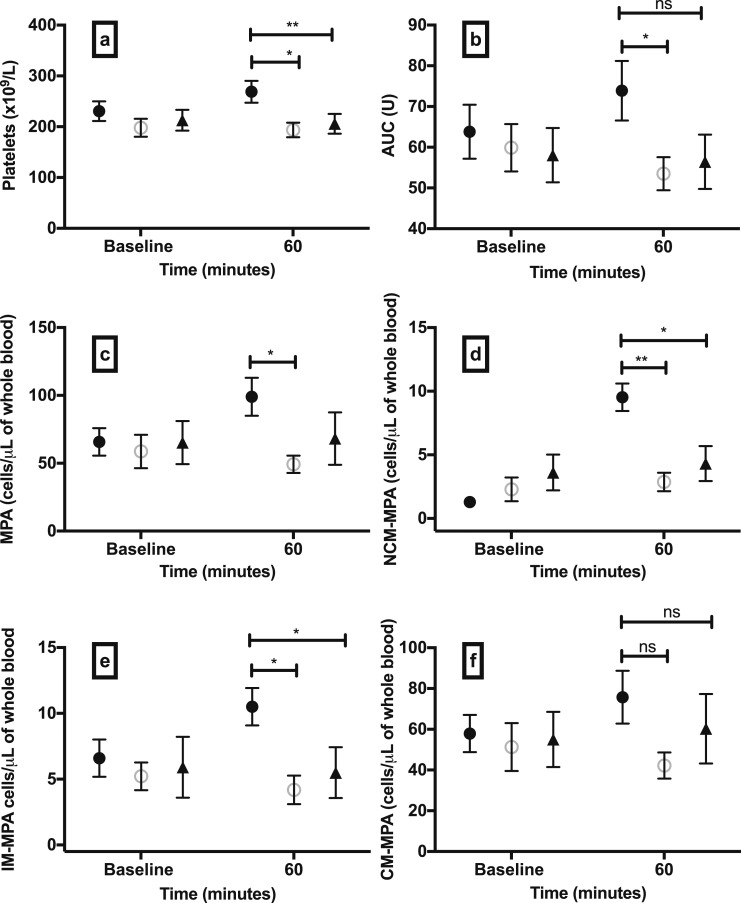
Platelet reactivity and MPA formation in experimental hypoglycemia and controls. (a) Total platelet count, (b) platelet aggregation to ADP 6.45 μM, (c) total MPA formation, and MPA formation within monocyte subsets (d) NCM-MPA, (e) IM-MPA, and (f) CM-MPA after 60 min of hypoglycemia, euglycemia, or sham-saline injection. Data are mean (SEM). *P < 0.05; **P < 0.01; ns, nonsignificant; *P* values are provided for comparison between study groups. Black circles, hypoglycemia group; open circles, euglycemia group; black triangles, sham-saline group.

Activation of platelets and generation of platelet-leukocyte aggregates contribute to leukocyte mobilization and inflammation in the vasculature (36). We therefore studied platelet number and function and their interaction with leukocytes. Total platelet count increased in hypoglycemia compared with euglycemia and sham-saline controls (Fig. 4a). ADP-induced platelet aggregation increased after 60 minutes of hypoglycemia vs euglycemia (*P* = 0.014), and numerically (but not statistically significantly) higher platelet aggregation was detected in the hypoglycemia group compared with the sham-saline group (*P* = 0.064) (Fig. 4b). The total number of MPAs increased after 60 minutes of hypoglycemia compared with euglycemia (Fig. 4c). Although total MPAs were not significantly higher in the hypoglycemia group compared with sham-saline controls at 60 minutes (Fig. 4c), we observed specific increases in NCM-MPAs and IM-MPAs (Fig. 4d and 4e). CM-MPAs appeared to increase after 60 minutes of hypoglycemia vs euglycemia and sham-saline, but this increase was not statistically significant (*P* = 0.054) (Fig. 4f).

#### Cell surface markers

To further explore the activation state of monocytes after hypoglycemia, we studied expression levels of chemokine receptor CX_3_CR1 and integrin CD11b. Hypoglycemia did not alter the expression of CX_3_CR1 or CD11b (Supplemental Fig. 1a and 1b).

### Endotoxin challenge

To determine whether previous hypoglycemia affected the subsequent response to a classic immune activator and thus to reveal whether hypoglycemia had any longer-lasting effects on the innate immune system, we performed a low-dose intravenous endotoxin challenge 48 hours after the hypoglycemic challenge in all subjects. Consistent with the low-dose model used, no fever or significant change in mean arterial blood pressure was recorded after endotoxin challenge across the study groups.

### Epinephrine, cortisol, and GH

**Figure 5.**
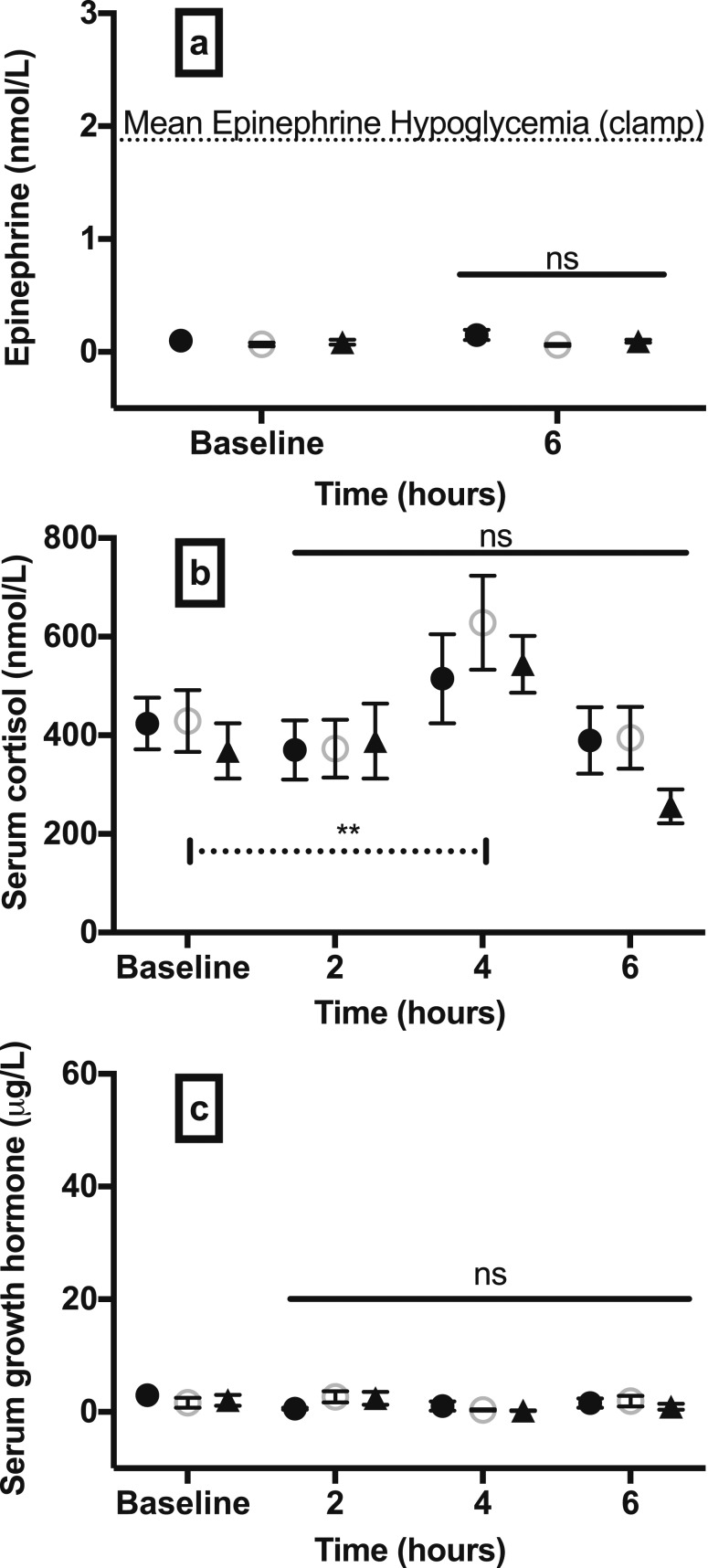
Changes in epinephrine, cortisol, and GH response after endotoxin challenge. (a) Epinephrine, (b) cortisol, and (c) GH responses 2, 4, and 6 h after low-dose (0.3 ng/kg) intravenous endotoxin challenge in participants who underwent hypoglycemia, euglycemia, or a sham-saline clamp 48 h earlier. Data are mean (SEM). **P < 0.01; ns, nonsignificant; *P* value on dashed line in (b) represents change in cortisol at 4 h compared with baseline in all groups; solid horizontal lines represent significance for comparison between study groups. Dashed line in (a) illustrates the mean epinephrine response in the hypoglycemia clamp group. Black circles, hypoglycemia group; open circles, euglycemia group; black triangles, sham-saline group.

In contrast to the stress response induced by hypoglycemia, epinephrine levels were not significantly different between study groups 6 hours after endotoxin administration (Fig. 5a). In the hypoglycemia group, epinephrine levels were 0.15 ± 0.04 nmol/L vs 0.06 ± 0.01 nmol/L in euglycemia group and 0.09 ± 0.01 nmol/L in sham-saline group. There were also no differences detected between groups in serum cortisol and GH levels after endotoxin administration (Fig. 5b and 5c). However, a rise compared with baseline in the stress hormone cortisol was evident whereby serum cortisol levels peaked at 4 hours after endotoxin challenge in all study groups (*P* = 0.005) (Fig. 5b).

### Total and differential leukocyte count

**Figure 6.**
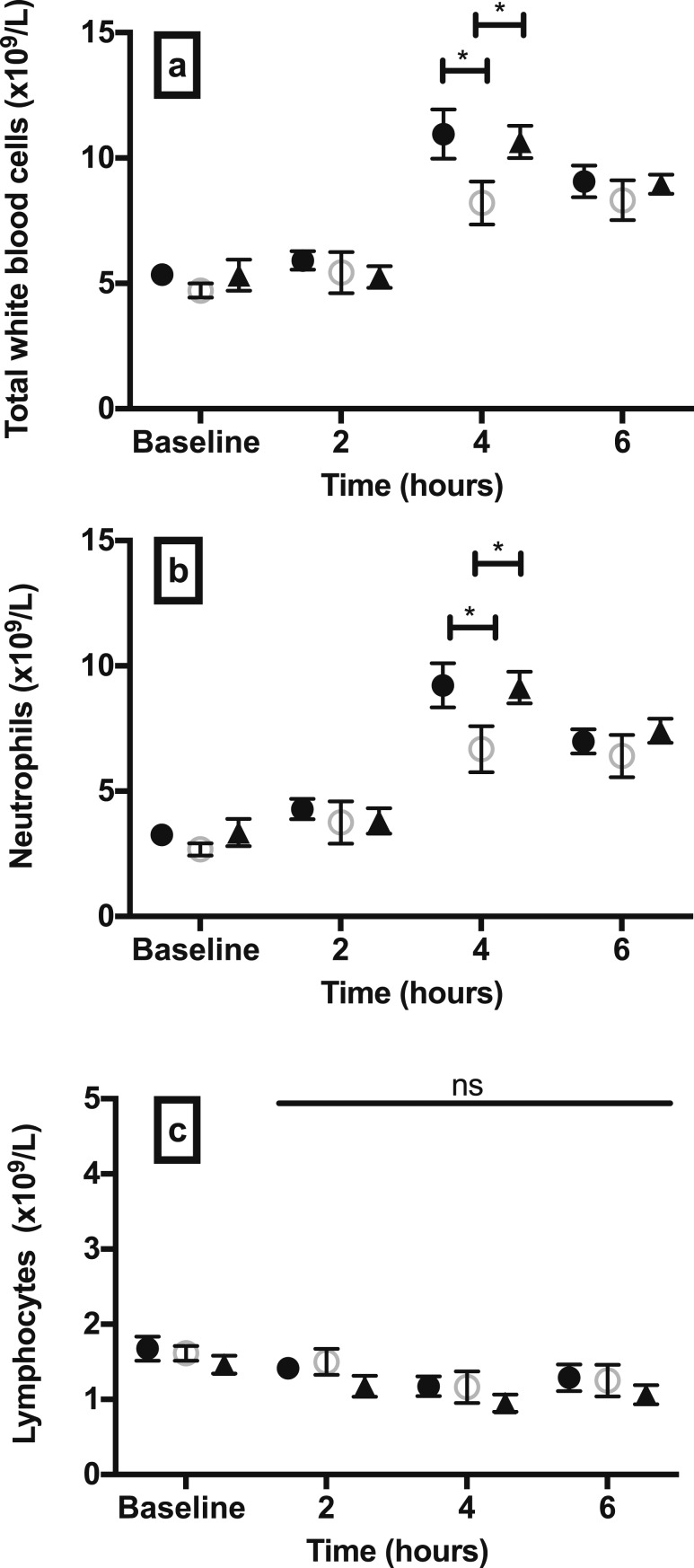
Peripheral total WBC, neutrophil, and lymphocyte kinetics after endotoxin challenge. Number of circulating (a) total WBCs, (b) neutrophils, and (c) lymphocytes 2, 4, and 6 h after low-dose (0.3 ng/kg) intravenous endotoxin challenge in participants who underwent hypoglycemia, euglycemia, or a sham-saline clamp 48 h earlier. Data are mean (SEM). *P < 0.05; ns, nonsignificant; *P* values are provided for comparison between study groups; solid horizontal line in (c) represents significance for comparison between study groups. Black circles, hypoglycemia group; open circles, euglycemia group; black triangles, sham-saline group.

**Figure 7.**
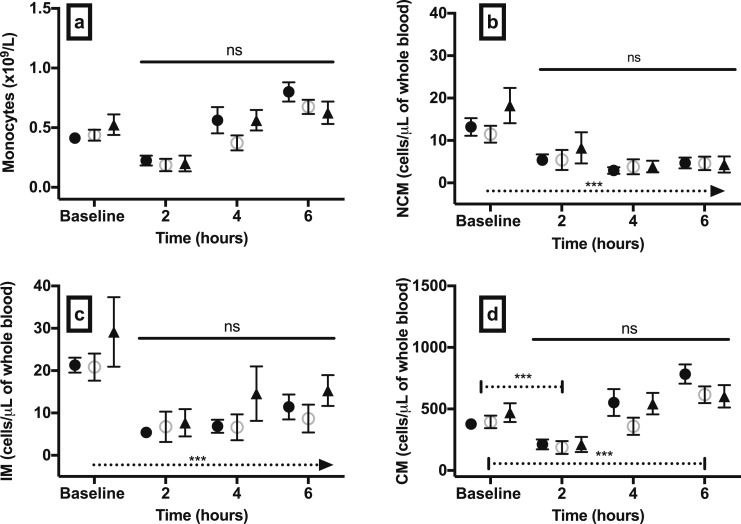
Total monocyte count and monocyte subset kinetics after endotoxin challenge. Absolute circulating numbers of (a) total monocytes and monocyte subsets (b) NCM, (c) IM, and (d) CM 2, 4, and 6 hours after low-dose (0.3 ng/kg) intravenous endotoxin challenge in participants who underwent hypoglycemia, euglycemia, or a sham-saline clamp 48 h earlier. Data are mean (SEM). ***P < 0.001; ns, nonsignificant; *P* value on dashed line in (b) represents change in number of NCMs at 2, 4, and 6 hours compared with baseline in all study groups. *P* value on dashed line in (c) represents change in number of IMs at 2, 4, and 6 h compared with baseline in all study groups. *P* values on dashed lines in (d) represent change in number of CMs at 2 and 6 h compared with baseline in all study groups. Solid horizontal lines represent significance for comparison between study groups. Black circles, hypoglycemia group; open circles, euglycemia group; black triangles, sham-saline group.

We observed that antecedent hypoglycemia modulated the subsequent WBC response to endotoxin. Total number of WBCs increased significantly after endotoxin in all study groups (Fig. 6a). The peak WBC response occurred at 4 hours after endotoxin, and this level was significantly higher in the hypoglycemia group at 10.96 ± 0.97 × 10^9^/L vs 8.21 ± 0.85 × 10^9^/L in the euglycemia group (*P* = 0.012) (Fig. 6a). Total WBC count 4 hours after endotoxin in the sham-saline group was 10.65 ± 0.64 × 10^9^/L, and this level was significantly higher than in the euglycemia group (*P* = 0.033) but not the hypoglycemia group (*P* = 0.974). The rise in WBC was mainly a consequence of an increase in neutrophil count (Fig. 6b). The lymphocyte count decreased after endotoxin (Fig. 6c), and the monocyte count initially decreased before recovery 6 hours after endotoxin (Fig. 7a). There was a trend toward a higher total monocyte count in the hypoglycemia group 4 hours after endotoxin compared with euglycemia, but this comparison did not reach statistical significance (*P* = 0.085). The absolute number of circulating monocyte subsets did not differ significantly between study groups (Fig. 7b, 7c, and 7d). NCM and IM numbers decreased significantly after endotoxin compared with baseline values in all groups (*P* < 0.001) (Fig. 7b and 7c). Compared with baseline, CM numbers significantly declined at 2 hours (*P* < 0.001), before rising and reaching a peak at 6 hours (*P* < 0.001) (Fig. 7d).

### Cell surface markers

We examined monocyte activation after endotoxin exposure by measuring cell surface marker CX_3_CR1 expression. Endotoxin administration caused a significant decline in expression of this marker across all study groups compared with baseline (*P* < 0.001) (Supplemental Fig. 2a). This decline was accompanied by an increase in the concentration of CX_3_C chemokine ligand 1 in plasma at 4 and 6 hours compared with baseline in all groups (*P* < 0.001) (Supplemental Fig. 2e). Activation of monocytes was also revealed by increased expression of CD11b expression at 4 and 6 hours after endotoxin compared with baseline in all groups (*P* < 0.001) (Supplemental Fig. 3a). In addition, the percentage of total monocytes that were positive for CD11b expression was higher in hypoglycemia group than in the euglycemia group at 2 hours after endotoxin (*P* = 0.007) (Supplemental Fig. 3b).

## Discussion

Hypoglycemia may contribute to exacerbations of ischemic CV disease. We aimed to investigate the effect of acute experimental hypoglycemia and subsequent low-dose endotoxemia on aspects of the innate immune response (total leukocytes, leukocyte subsets, and specifically monocyte subsets), thrombosis (platelet aggregation), and crosstalk between inflammation and thrombosis (MPAs). Our main findings were that hypoglycemia increased the number of all three circulating monocyte subsets, in association with a stress response characterized by increased plasma epinephrine levels; hypoglycemia increased platelet reactivity, promoted formation of MPAs, and promoted aggregate formation between proinflammatory monocytes and platelets; leukocyte mobilization to the stress response of low-dose endotoxin was independent of epinephrine; and antecedent hypoglycemia resulted in a significantly higher inflammatory leukocyte response to low-dose endotoxin administered 48 hours later.

As shown previously (13, 37), we confirm that hypoglycemia results in leukocytosis. In addition, we present the effect of hypoglycemia on monocyte subset kinetics and demonstrate an increase in the absolute number of all three circulating monocyte subsets. The largest increase was observed in numbers of circulating NCMs (twofold) and IMs (1.8-fold), with a modest increase in the number of CMs (1.3-fold). These data are in keeping with an observed selective mobilization of CD16^+^ monocytes in response to exercise (27, 38) and epinephrine infusion (39). Ratter *et al.* (37) also recently determined that hypoglycemia might modify selective monocyte mobilization. However, they did not phenotype monocyte subsets but rather measured total levels of CD16 on peripheral blood mononuclear cells isolated from both healthy participants and those with type 1 diabetes in experimental hypoglycemia settings. Our data identify specific changes in monocyte subsets that have been previously linked to monocyte activation and atherogenesis. Because observational data support the notion of CD16^+^ monocytes being proatherogenic (20, 22–24), and adrenergic modulation of monocytes induces proinflammatory changes (40), an increase in the circulating number of these cells after hypoglycemia may increase CV risk in diabetes.

Previous studies investigating effects of hypoglycemia on platelet biology have suggested an increase in platelet reactivity; however, this increase was in the context of hypoglycemic stimulus as part of an insulin stress test (41). An older investigation into the effect of hypoglycemia on monocyte-platelet interactions in type 1 diabetes and healthy controls also suggested a trend toward increased MPA formation, but these data were not conclusive, with little difference between euglycemic and hypoglycemic conditions (42). Our study also recapitulates and extends previous findings that hypoglycemia is prothrombotic, as evidenced by an increased platelet count and increased platelet reactivity to ADP (43). We have conclusively demonstrated an overall increase in formation of MPAs in hypoglycemia in comparison with euglycemia. Furthermore, we provide data demonstrating MPA formation within monocyte subsets in experimental hypoglycemia. MPA formation is a highly sensitive marker of both monocyte and platelet activation (44, 45). MPA formation promotes monocyte release of the proinflammatory cytokines TNF*α*, C-X-C motif chemokine ligand 8, and C-C motif chemokine ligand 2 (46, 47) and increases adhesive properties of monocytes (48), thereby representing a bridge between inflammation and thrombosis that may increase CV risk. In acute coronary syndromes, MPA formation correlates with troponin elevation, risk of in-hospital cardiac events including death, and risk of future cardiac events (20, 49). We have also shown that NCMs and IMs aggregate more readily with platelets in response to hypoglycemia compared with CMs. A similar observation of proportionally higher IM-MPA and NCM-MPA formation has been reported in patients after an ST elevation myocardial infarction, with higher IM-MPAs in particular being a poor prognostic indicator at 6 weeks after ST-elevation myocardial infarction (20). Thus, our data suggest that hypoglycemia not only increased circulating numbers of CD16^+^ monocytes but also promoted increased interaction between these proinflammatory monocyte subsets and platelets.

We wanted to determine whether antecedent hypoglycemia modulated responses to low-dose endotoxin. We chose a low-dose endotoxin model because we thought it was the safest way to combine the clamp and endotoxin human models, because future extension to the study of human diabetes would be more feasible with this model, and because patients with diabetes are often exposed to chronic low-grade infections through foot ulceration and periodontitis, which might further increase the risk of CV mortality (50, 51). In our model, we observed in all groups that monocytes were activated even in response to low-dose endotoxin, as indicated by upregulation of systemic levels of the CX_3_CR1 ligand, CX_3_C chemokine ligand 1, and upregulation of the adhesion molecule CD11b on the monocytes themselves. Interestingly, compared with euglycemia, hypoglycemia resulted in greater leukocyte mobilization in response to low-dose intravenous endotoxemia 48 hours later. Furthermore, we noted a nonsignificant trend toward a higher total monocyte count in the hypoglycemia group 4 hours after endotoxin compared with euglycemia. The percentage of monocytes that were CD11b positive was also higher in the hypoglycemia group compared with the euglycemia group at 2 hours after endotoxin. Levels of leukocyte mobilization were similar between groups who received previous sham-saline or hypoglycemia. These data suggest that euglycemia with insulin suppressed leukocyte mobilization in response to endotoxin 48 hours later, consistent with the known anti-inflammatory actions of insulin (52, 53), and that the physiological stress of hypoglycemia overcame this insulin-mediated suppression of inflammatory responses. Our data show that drivers for differential leukocyte mobilization to endotoxin are unlikely to be due to differences between groups in epinephrine, cortisol, and GH levels after endotoxin because these levels were not significantly different. Our observation that a single episode of hypoglycemia compared with euglycemia invokes a stronger proinflammatory response to endotoxin up to 2 days later is of potential clinical relevance given that trial data suggest downstream mortality after hypoglycemia (29–31).

The strengths of our study include use of a human experimental model and detailed flow cytometric analysis that allowed us to comprehensively describe immune cell kinetics and activation status in response to experimental hypoglycemia and endotoxin challenge *in vivo*. The separation of clamp and endotoxin studies by 48 hours allowed us to probe the longitudinal effects of hypoglycemia on innate immunity. Moreover, by using a sham-saline group, we specifically controlled for the immunological effects of insulin, thereby robustly investigating proinflammatory changes in response to hypoglycemia.

One limitation was our decision to study a small number of young, healthy participants, which limits the applicability of our findings to older patients with diabetes, established CV risk factors, and atherosclerosis. For ethical and safety reasons, we decided to examine our experimental model initially in healthy participants. We also specifically adopted a low-dose endotoxin model, with future translatability in older, higher-risk participants in mind. Future studies should therefore confirm our findings in patients with diabetes. In addition, it is worth noting that we studied cell numbers, phenotypic changes, and activation status in circulating immune cells, and these data may not necessarily reflect the functional capacity of these cells in an atherosclerotic plaque. An animal model of combined experimental hypoglycemia and atherosclerosis may help resolve these questions.

In conclusion, hypoglycemia mobilized proatherogenic monocyte subsets and induced prothrombotic changes by increasing platelet reactivity. In addition, hypoglycemia amplified interactions between platelets and monocytes by promoting MPA formation with increased aggregation of proinflammatory monocytes with platelets. Hypoglycemia may also prime the innate immune system to respond more robustly to stimuli such as endotoxin. This finding implies proinflammatory consequences of hypoglycemia beyond the acute episode. These data provide mechanistic insights into how hypoglycemia could increase CV risk through upregulation of inflammatory responses.

## Supplementary Material

Supplemental Figure

## Abbreviations:

CM: classic monocyte
CV: cardiovascular
CX_3_CR1: CX_3_C chemokine receptor 1
FACS: fluorescence-activated cell sorting
IM: intermediate monocyte
MPA: monocyte-platelet aggregate
NCM: nonclassic monocyte
PE: phycoerythrin
WBC: white blood cell
