# Supplementary material for: Effect of Hypoglycemia on Inflammatory Responses and the Response to Low-Dose Endotoxemia in Humans
Source: J Clin Endocrinol Metab. 2018 Sep 24;104(4):1187–99. doi: 10.1210/jc.2018-01168 (PMC6391720; doi:10.1210/jc.2018-01168)
Supplement: Supplemental Figure [file jc.2018-01168.sf1.pdf]

Supplemental data

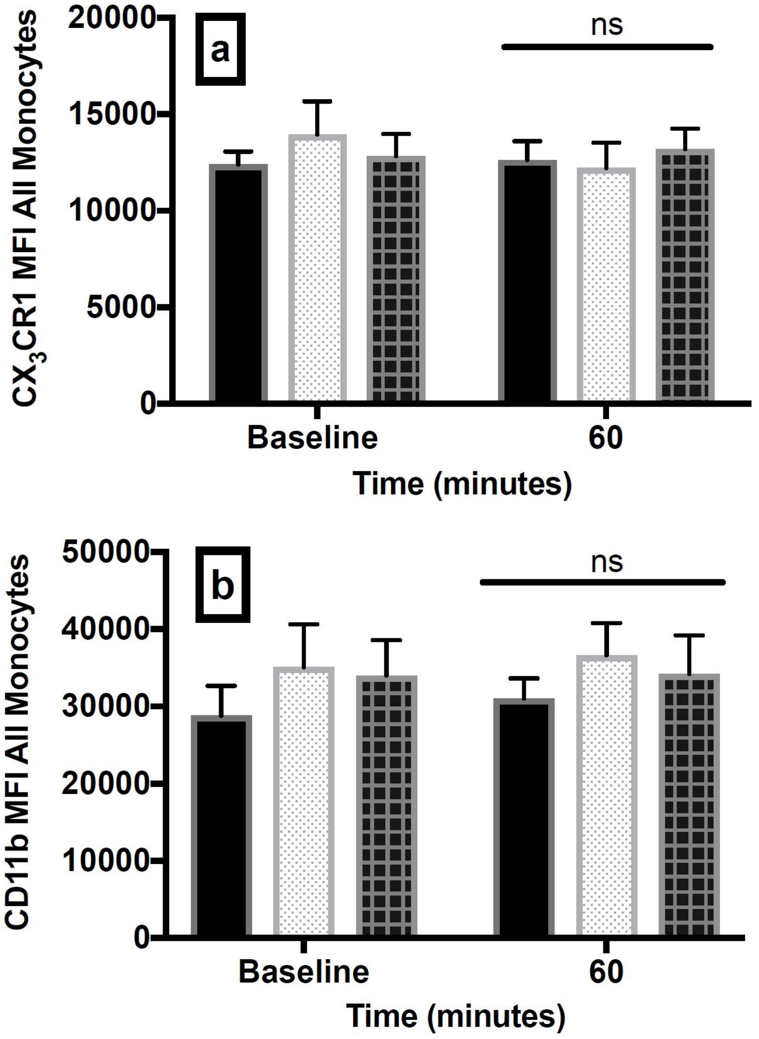

Supplemental Figure 1: Expression of CX_3CR1 and CD11b on monocytes in clamp studies. Expression of CX_3CR1 (a) and CD11b (b) on all monocytes after 60 minutes of hypoglycemia, euglycemia or sham-saline injection. Data are geometric mean (SEM), ns-non-significant, solid horizontal lines represent significance for comparison between study groups. Black bars-hypoglycemia group; white bars-euglycemia group; striped black bars-sham-saline group. MFI, mean fluorescence intensity.

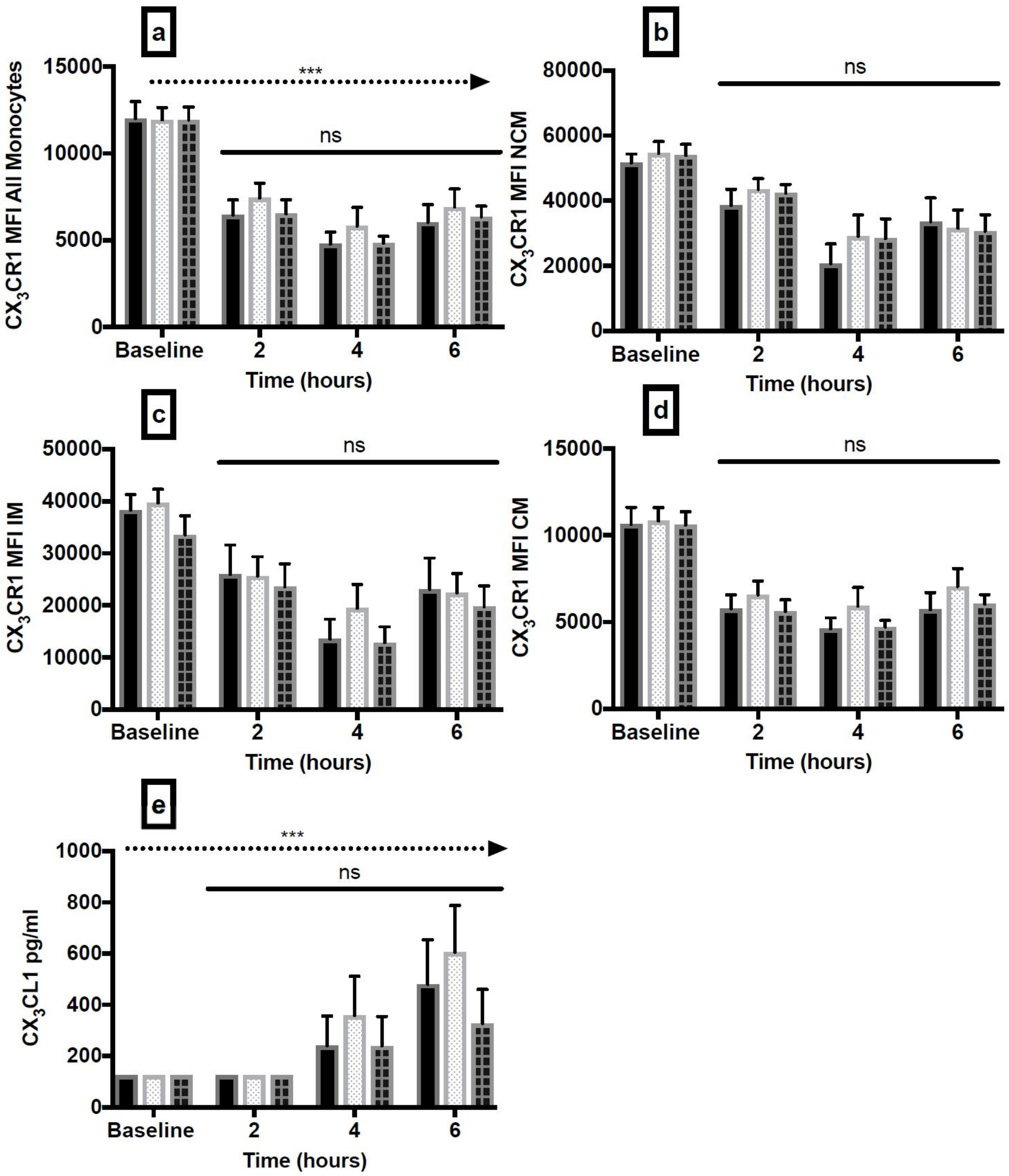

Supplemental Figure 2: Expression of CX_3CR1 on monocytes and plasma levels of CX_3CL1 post endotoxin challenge. CX_3CR1 expression on total monocytes (a) and monocyte subsets comprising of NCM (b), IM (c) and CM (d) 2, 4 and 6 hours following low dose (0.3 ng/kg) intravenous endotoxin challenge in participants that underwent hypoglycemia, euglycemia or a sham-saline clamp 48 hours earlier. CX_3CL1 values determined in plasma are shown in (e). Data are geometric mean (SEM), ***$P<0.001$, ns-non-significant, P-values on dashed line in (a) and (e)

represent changes in CX_3CR1 on all monocytes and plasma CX_3CL1 expression respectively at 2, 4 and 6 hours compared to baseline in all study groups. Solid horizontal lines represent significance for comparison between study groups. Black bars-hypoglycemia group; white bars-euglycemia group; striped black bars-sham-saline group. NCM, non-classical monocytes; IM, intermediate monocytes; CM, classical monocytes; MFI, mean fluorescence intensity.

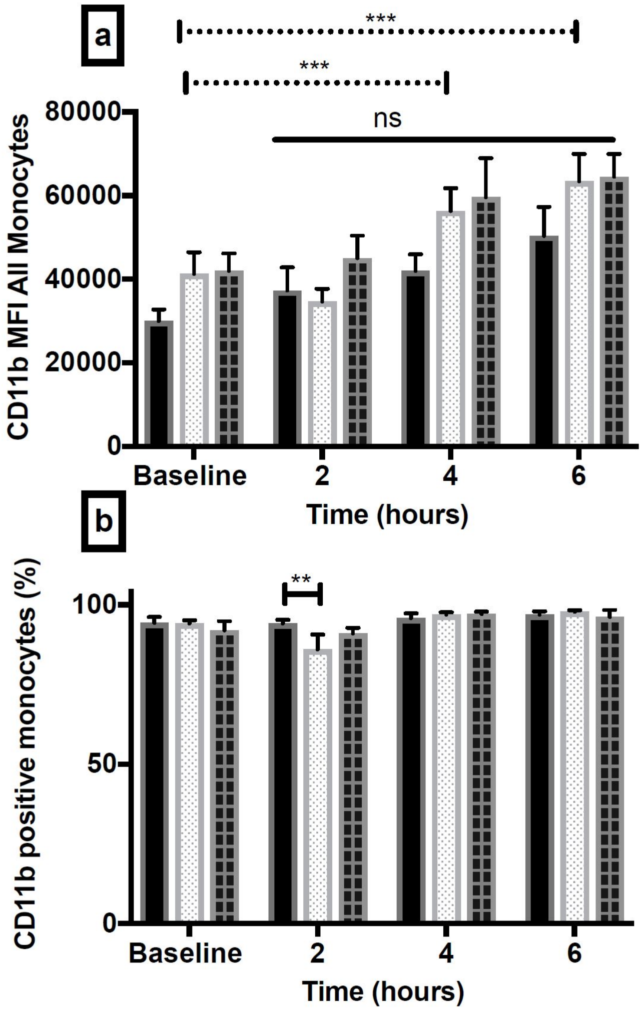

Supplemental Figure 3: Expression of CD11b on monocytes and percentage of CD11b positive monocytes post endotoxin challenge. CD11b expression on total monocytes (a) and percentage (%) of monocytes that positively express CD11b (b) 2, 4 and 6 hours following low dose (0.3 ng/kg) intravenous endotoxin challenge in participants that underwent hypoglycemia, glycaemia or a sham-saline clamp 48 hours earlier. Data are geometric mean (SEM) in (a) and mean (SEM) in (b), **P<0.01, ***P<0.001, ns-non-significant, dashed lines in (a) represents change in CD11b expression on all monocytes at 4 and 6 hours compared to baseline in all study groups. P-value in (b) is for comparison of CD11b percentage expression between hypoglycemia and glycaemia. Solid horizontal line in (a) represents significance for comparison between study groups. Black bars-hypoglycemia group; white bars-

euglycemia group; striped black bars-sham-saline group. MFI, mean fluorescence intensity.

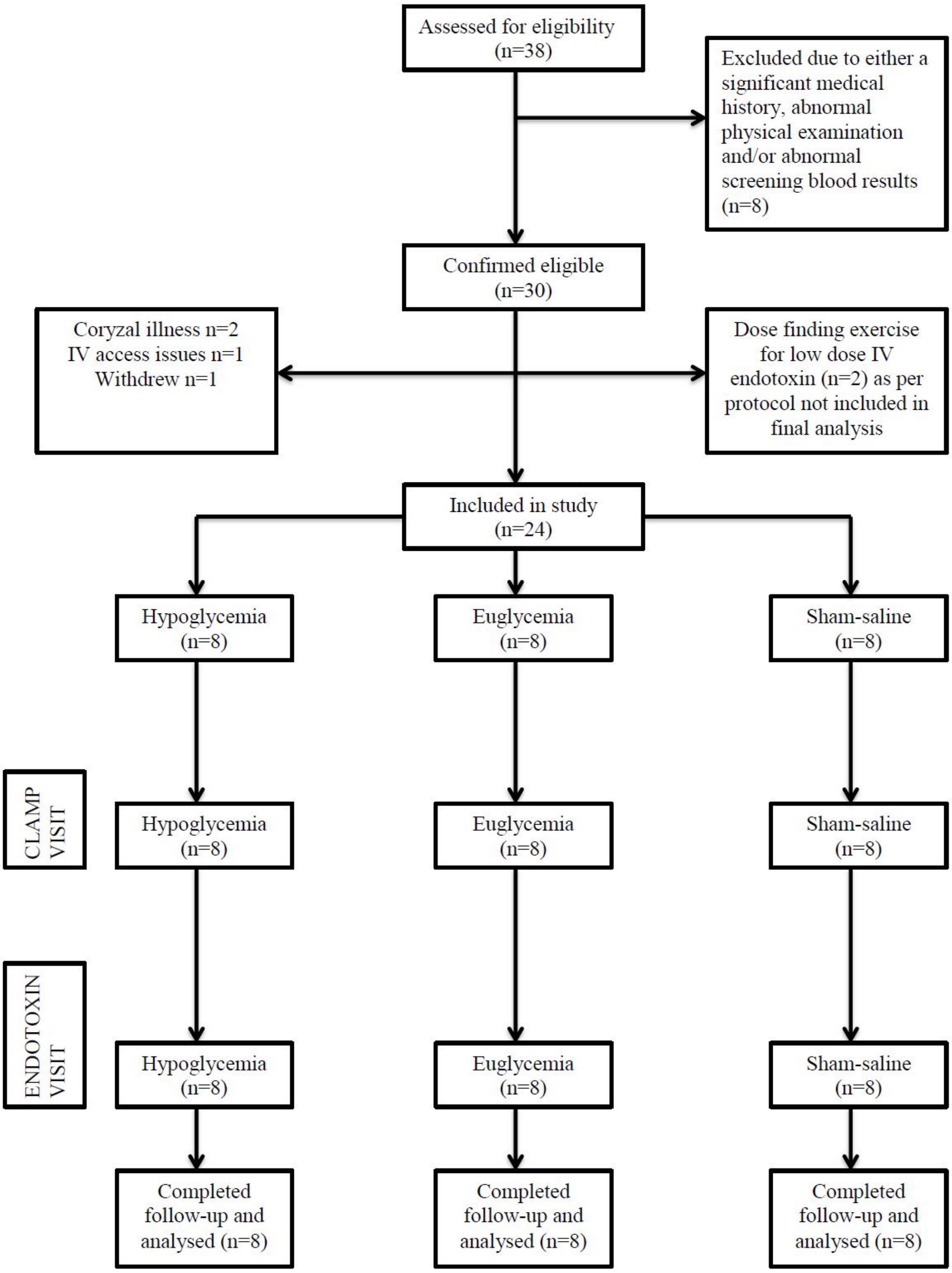

Supplemental flow diagram: Participant screening, exclusions, recruitment, follow-up and analysis.
